# Molecular Diversity of Eukaryotes in Municipal Wastewater Treatment Processes as Revealed by 18S rRNA Gene Analysis

**DOI:** 10.1264/jsme2.ME14112

**Published:** 2014-12-10

**Authors:** Kengo Matsunaga, Kengo Kubota, Hideki Harada

**Affiliations:** 1 Department of Civil and Environmental Engineering, Tohoku University, 6–6–06 Aza-Aoba, Aramaki, Aoba-ku, Sendai, Miyagi 980–8579, Japan

**Keywords:** 18S rRNA gene, Alveolata, eukaryotic community composition, Fungi, municipal wastewater treatment

## Abstract

Eukaryotic communities involved in sewage treatment processes have been investigated by morphological identification, but have not yet been well-characterized using molecular approaches. In the present study, eukaryotic communities were characterized by constructing 18S rRNA gene clone libraries. The phylogenetic affiliations of a total of 843 clones were Alveolata, Fungi, Rhizaria, Euglenozoa, Stramenopiles, Amoebozoa, and Viridiplantae as protozoans and Rotifera, Gastrotricha, and Nematoda as metazoans. Sixty percent of the clones had <97% sequence identity to described eukaryotes, indicating the greater diversity of eukaryotes than previously recognized. A core OTU closely related to *Epistylis chrysemydis* was identified, and several OTUs were shared by 4–8 libraries. Members of the uncultured lineage LKM11 in Cryptomycota were predominant fungi in sewage treatment processes. This comparative study represents an initial step in furthering understanding of the diversity and role of eukaryotes in sewage treatment processes.

Sewage treatment processes can be considered as artificial ecosystems, in which eukaryotes are key microbial components. Protozoans and metazoans are often used as indicators of treatment performance. Eukaryotes are the primary predators of prokaryotes; their predation of dispersed bacteria improves sludge sedimentation and effluent water quality in terms of turbidity, biological oxygen demand (BOD), and suspended solids (27), and also decreases the risk of exposure to bacterial pathogens (28). Some Rotifera lineages specifically graze on filamentous fungi that are known to cause bulking in activated sludge (10), and these organisms can, therefore, be used to prevent sludge bulking. Furthermore, recent studies reported the useful implementation of fungi for granular formation in sewage treatment processes (35) as well as the degradation of cellulose, hemicellulose, and lignin biomass (7). Fungi are known to contribute to denitrification, and some can grow under various O_2_ conditions (concentrations) through three different energy-yielding metabolic pathways: O_2_ respiration, denitrification (nitrite respiration), and ammonia fermentation (13). Some eukaryotes belonging to the Rhizaria have been reported to accumulate nitrate under aerobic conditions and to respire using this accumulated nitrate through denitrification under anoxic conditions (31). Due to these important processes, the contribution of eukaryotes to denitrification in ocean sediments is estimated to be equal to that of bacteria (29); thus, eukaryotes may also play a significant role in the removal of carbon and nitrogen in sewage treatment processes.

The eukaryotes involved in sewage treatment processes have traditionally been identified morphologically and enumerated by microscopic observations. However, morphological identification is often hampered due to limited or difficult diagnostic criteria for some taxa and the time-consuming processes necessary to acquire identification expertise. *Minidiscus* cells are easily overlooked because of their small size (1.9–7.5 μm) (25). Furthermore, the functional identification of eukaryotes requires that they are isolated, which can be difficult, especially for small eukaryotes. Thus, the role played by eukaryotic species in many environments—including wastewater treatment processes—remains unclear.

These limitations have driven the development of alternative (*i.e.*, molecular) identification methods. The phylogenetic diversity of eukaryotes has been investigated by the construction of 18S rRNA gene clone libraries or tag pyrosequencing methods (3, 25, 33). The molecular diversity of eukaryotes in activated sludge sewage treatment processes has also been investigated (8, 9, 23, 26, 27, 36). These studies investigated specific members of eukaryotes (*e.g.*, Ciliophora and Fungi) or eukaryotes in lab-scale reactors with a small number of clones. Thus, there have been no comprehensive investigations of eukaryotic molecular diversity in full-scale sewage treatment processes, and the diversity and roles of eukaryotes there consequently remain unclear.

Eukaryotic communities in sewage treatment processes are influenced by the type of process and operating conditions (6). Various processes are employed to treat sewage, and each full-scale sewage treatment plant receives different amounts and concentrations of sewage, resulting in different operational conditions. Therefore, the eukaryotic compositions of full-scale sewage treatment plants are expected to be highly diverse, and previously unrecognized eukaryotes may be present and play important roles. We here investigated the eukaryotic communities of 9 sludge samples collected from 3 different full-scale sewage treatment processes (activated sludge [AS], anoxic/oxic activated sludge [AO], and oxidation ditch [OD]) by constructing 18S rRNA gene clone libraries. We identified core and shared eukaryotes in sewage treatment processes. Furthermore, the results obtained showed that sewage treatment processes can be characterized by a greater diversity of uncultured eukaryotes than was previously recognized.

## Materials and Methods

### Sludge samples

Sludge samples were taken from 3 different sewage treatment processes (*i.e.*, AS, AO, and OD) at 5 different sewage works. All sewage works were located in Japan and operated without temperature control. Four AS samples, designated AS_N_Sep, AS_N_Dec, AS_S_Dec, and AS_K_Jan, were collected from aeration tanks at 3 sewage treatment plants. AS_N samples were collected twice in different months (September and December). Samples from a two-step AO process were also collected twice from a first anoxic tank (AO_an_Mar and AO_an_Dec) and a first aeration tank (AO_ox_Mar and AO_ox_Dec) in March and December. An OD sample (OD_Dec) was collected in December. The sampling points of AS and AO samples were located between the influent point and center of each tank while that of an OD sample was very near to the aeration area of the tank.

### Operational parameters and water quality analysis

Data regarding the average flow rate, hydraulic retention time (HRT), sludge retention time (SRT), and BOD for each sampling month were supplied by the respective sewage work, whereas the other parameters were measured using a grabbed sample. Dissolved oxygen (DO) was measured on site using a DO meter (Multi 3430, WTW, Weilheim, Germany). Samples filtered with a glass fiber filter (0.4 μm, GB140, ADVANTEC, Tokyo, Japan) were used to determine total organic carbon (TOC), chemical oxygen demand (COD), total nitrogen (T-N), ammonium-nitrogen (NH_4_^+^-N), nitrate-nitrogen (NO_3_^−^-N), nitrite-nitrogen (NO_2_^−^-N), total phosphorus (T-P), and phosphate-phosphorus (PO_4_^3−^-P). TOC was determined using a TOC-L analyzer (Shimadzu, Kyoto, Japan). COD was determined using COD digestion vials (low range, HACH, Loveland, CO, USA) and a DR2500 spectrophotometer (HACH). T-N and T-P were measured using an Auto Analyzer II (BLTEC, Tokyo, Japan). NH_4_^+^-N, NO_3_^−^-N, NO_2_^−^-N, and PO_4_^3−^-P were measured using a QuAAtro 2-HR (BLTEC). Mixed liquor suspended solids (MLSS) and mixed liquor volatile suspended solids (MLVSS) of sludge retained in the reactors were determined as prescribed in the standard method (1). Details regarding operation parameters and process performance are shown in Table 1.

### DNA extraction, cloning of rRNA genes, and phylogenetic analysis

Sludge samples were washed with phosphate-buffered saline (137 mM NaCl, 8.10 mM Na_2_HPO_4_, 2.68 mM KCl, 1.47 mM KH_2_PO_4_ [pH 7.4]) immediately after sampling. DNA was extracted from approximately 0.3–0.5 g (wet-weight) of sludge samples using an ISOIL for the Beads Beating kit (NIPPON GENE, Tokyo, Japan).

Near-full-length eukaryotic 18S rRNA genes were amplified using the EukA (5′-AAC CTG GTT GAT CCT GCC AGT-3′) and EukB (5′-TGA TCC TTC TGC AGG TTC ACC TAC-3′) universal eukaryotic primer set (23) and *TaKaRa Ex Taq* Hot Start Version (TaKaRa). The PCR conditions were as follows: initial denaturation at 98°C for 20 s, followed by a variable number of cycles at 98°C for 10 s, 53°C for 30 s, and 72°C for 120 s, with final extension at 72°C for 7 min. The number of PCR cycles was optimized for each sample in the range of 17 to 27. After purification with a MinElute PCR Purification Kit (Qiagen, Tokyo, Japan) or High Pure PCR Product Purification Kit (Roche), the PCR products were cloned using a TOPO TA Cloning Kit (Life Technologies, Carlsbad, CA, USA). Between 62 and 111 clones from each sample (834 clones in total) were partially sequenced with EK-555F (5′-AGT CTG GTG CCA GCA GCC GC-3′) (20) (approximately 600 nt), aligned, and classified into operational taxonomic units (OTUs) with a threshold value of 0.03 (corresponding to a sequence identity ≥97%) using mothur (32). The near-full-length sequences (*ca.* 1,800 nt) of representative clones from each OTU were determined (142 clones in total). Clones that were not sequenced well with the EK-555F primer (*i.e.*, primer mismatch and/or sequencing errors) were sequenced using the M13f or M13r primer. The sequences were subjected to BLAST searching (http://blast.ncbi.nlm.nih.gov/Blast.cgi) using the National Center for Biotechnology Information (NCBI) database. Phylogenetic analyses were conducted using ARB software (21) with the SILVA database, release 115 (30). The diversity and richness of the eukaryotic assemblages were estimated as previously described (16). In brief, the diversity and richness of the eukaryotic assemblages were estimated using the Chao 1 and Shannon-Wiener indices, as calculated using EstimateS, version 8.2.0 (http://viceroy.eeb.uconn.edu/EstimateS). Coverage was calculated using the equation (1−[n/N]), where n represents the number of OTUs represented by a single clone, and N represents the total number of clones retrieved. The evenness index was determined using the equation H/ln R, where H represents the Shannon-Wiener index and R represents the number of OTUs observed. Gene sequence data were deposited in the GenBank/EMBL/DDBJ databases under accession numbers AB901525 to AB902358.

## Results and Discussion

### Operational properties of wastewater treatment plants

The operational conditions and performance characteristics of each plant and the water quality measures for the 9 sludge samples are shown in Tables 1 and S1. The performance of all plants was effective and stable under controlled conditions. The SRT of the OD and AO process was calculated over 30 d because this process involved a higher sludge circulation rate (114% in OD, 47–72% in AO). The organic loading rates (OLRs) were between 0.08 and 0.69 kgBOD m^−3^ d^−1^. The DO was approximately 1 mg L^−1^ or less in the samples.

### Overall eukaryotic community structure

The relative abundances of the 834 clones at the kingdom/superphylum level are shown in Fig. S1, and a total of 80 OTUs were obtained after aligning and clustering all samples with a 97% sequence identity threshold. Forty-one percent of all clones were classified within the Alveolata, and 99% of these sequences belonged to the Ciliophora, which are common organisms in sewage treatment processes. In addition to the Alveolata, other major eukaryotic phyla in the sewage treatment process samples were Fungi and Rhizaria, which accounted for 33 and 11% of the sequences, respectively. The Metazoa accounted for 11% of all clones.

Sequences were then further separated into two groups: described and uncultured groups. The described group included clones showing ≥97% sequence identity to the described eukaryotes, whereas the uncultured group included clones with <97% sequence identity to the described eukaryotes. The described group contained 331 sequences, whereas the uncultured group contained 503 sequences (60% of all clones) (Fig. S1). All Rhizaria and Metazoa sequences were in the described group. The uncultured group contained sequences of organisms belonging to the Alveolata (49%), Fungi (46%), Euglenozoa (4%), and other groups (1%). Uncultured organisms represented 15–89% of the sequences in each library (Fig. S2).

### Eukaryotic communities in each treatment process

Eukaryotic 18S rRNA gene clone libraries were constructed from 9 samples (Table 2). Between 62 and 111 clones from each sample (834 clones in total) were partially sequenced, and 10–21 OTUs were retrieved from each sample by aligning and clustering independently with a 97% sequence identity threshold. Near-full-length sequences (*ca.* 1,800 bp) of a representative clone from each OTU (a total of 142 clones) were determined and subjected to phylogenetic analysis. Relatively high coverage values (0.83–0.97, Table 2) indicated that the number of clones was sufficient to estimate the eukaryotic communities. The Chao1 nonparametric estimator indicated that there should be 15–49 OTUs in the samples (Table 2). The evenness indices for the AS_S_Dec, AS_K_Jan, and AO_an_Mar samples were low, whereas the coverage indices of these samples were higher. The composition of the eukaryotic community at the kingdom/superphylum level for each library and the list of OTUs are shown in Tables 2 and S2, respectively. Protozoa, primarily belonging to the Alveolata and Fungi, dominated in all libraries, whereas higher fractions of metazoans were found in the AS_N_Sep and OD_Dec libraries.

**Fig. 1:**
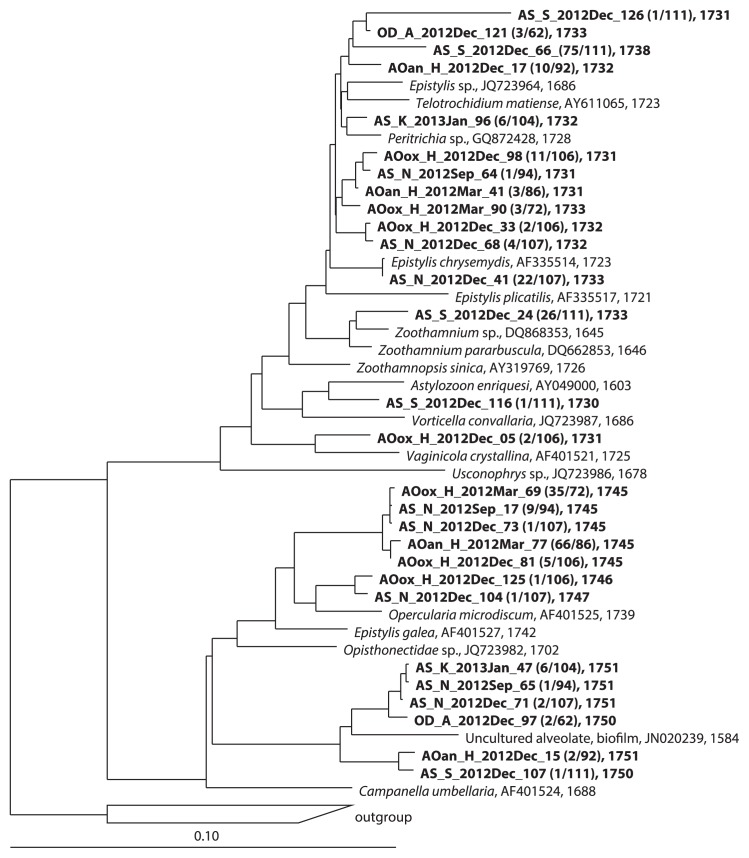
Phylogenetic tree of the subclass Peritrichia in the Alveolata based on a comparative analysis of 18S rRNA gene sequences. The tree shows the phylogenetic positions of the clones obtained in this study.

**Fig. 2:**
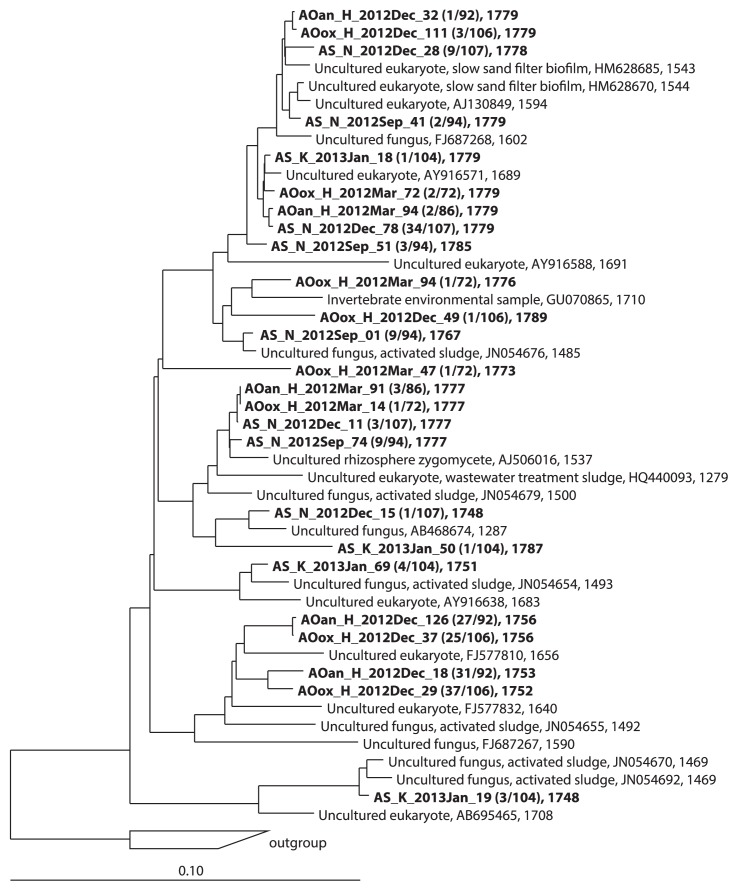
Phylogenetic tree of fungi in the LKM11 lineage based on a comparative analysis of 18S rRNA gene sequences. The tree shows the phylogenetic positions of the clones obtained in this study.

The predominant members in the AS samples were Alveolata in the AS_S_Dec library, Rhizaria, Alveolata, and Fungi in the AS_K_Jan library, Fungi, Alveolata, and Nematoda in the AS_N_Sep library, and Fungi, Alveolata, and Rotifera in the AS_N_Dec library (for the list of OTUs, see Table S2). In the AS_S_Dec library, 2 OTUs in the subclass Peritrichia were dominant (Fig. 1): 1 OTU (75/111 clones) clustered with *Epistylis chrysemydis* (accession number: AF335514), *Telotrochidium matiense* (accession number: AY611065) and *Peritrichia* sp. (accession number: GQ872428), and another OTU (26/111 clones) were found to be closely related to a *Zoothamnium* sp. (accession number: DQ868356). In the AS_K_Jan library, the relative abundance of a single OTU closely related to a *Rhogostoma* sp. (accession number: HQ121436) belonging to the Rhizaria was significantly high (80/111 clones). The AS_N_Sep and AS_N_Dec samples were taken from the same reactor, but the water temperatures at the time of sampling were significantly different: 28.0°C in September and 7.7°C in December. OTUs belonging to the uncultured fungal lineage LKM11 were frequently found in both libraries (4 OTUs, 23/94 clones in the AS_N_Sep library and 4 OTUs, 47/107 clones in the AS_N_Dec library, Fig. 2 and Table S2). The abundance of some OTUs fluctuated between the libraries. The frequency of a dominant OTU closely related to the nematode *Tobrilus gracilis* (accession number: AJ966506) decreased between September (37/94 clones) and December (2/107 clones). In contrast, OTUs closely related to *E. chrysemydis* (accession number: AF335514) and *Lepadella rhomoboides* (accession number: DQ297702) were found more frequently in December than in September (22/107 vs. 0/94 clones for *E. chrysemydis* and 11/107 vs. 1/94 clone for *L. rhomoboides*). The dominant group in December may be resistant to low temperatures. A previous study also indicated that the abundance of *Epistylis* sp. peaked in autumn, winter, and spring in AS treatment processes (5).

Most of the sequences retrieved from the OD sample belonged to Alveolata, Gastrotricha, and Fungi. In Alveolata, the most dominant member was affiliated to the class Phyllopharyngea unlike other sludge samples in which the subclass Peritrichia was abundant. Furthermore, the composition of Fungi differed from others: clones belonging to the uncultured LKM11 lineage were not detected, and Ascomycota and Basidiomycota were dominant fungi in the sludge samples. The plant received sewage containing hot spring water, and this factor, in combination with differences in this treatment process, may be one of the reasons for the establishment of such a unique community.

In the AO process, samples were taken twice from the same tanks in March and December, and, thus, a total of 4 AO libraries were constructed. The water temperature at the sampling point was 13.3°C in March and 18.5°C in December. The clone libraries were similar within the same sampling day, but differed between sampling months (March vs. December). The similarity may have been due to the relatively high sludge circulation rate (47–72%) of this process, whereas the difference may have been because of the water temperature. The relative abundances of the dominant OTUs differed in March and December: OTUs belonging to the subclass Peritrichia were more dominant in March than in December. OTUs belonging to the uncultured fungal lineage LKM11 predominated in December.

### Common eukaryotes in municipal wastewater treatment processes

The numbers of shared OTUs and sequences were plotted versus the number of libraries sharing each OTU (Fig. S3) in order to determine the shared and specific eukaryotes present in multiple different samples. The eukaryotes in this cluster can be considered core constituents. Eleven OTUs were shared among 4–8 libraries. The remaining 69 OTUs were specific to 1 library or shared by 2 libraries, and almost half of the sequences (430 sequences, corresponding to 51% of the total sequences) belonged to this group.

The eukaryotic OTUs shared among more than 4 libraries and their fractions in each library were shown in Table 3. The presence of shared OTUs indicated that aerobic municipal wastewater treatment processes involved the same basic populations despite differences in location, sewage characteristics, and operation parameters. Most of the shared OTUs, which included members of the Fungi, Alveolata, Stramenopiles, Rhizaria, and Rotifera, represented less than 10% of each library. In these, an OTU closely related to *E. chrysemydis*, which is in the subclass Peritrichia, was shared among 8 libraries (except for AS_S_Dec) (Table 3, No. VI). In addition to this core OTU, a total of 302 clones (36%) belonged to this subclass (Fig. 1). Eukaryotes belonging to this subclass clearly play an important role in sewage treatment processes, and the molecular survey indicated that activated sludge contained a high diversity of eukaryotes from the subclass Peritrichia. Eukaryotes belonging to this subclass (*e.g.*, the member of the genus *Epistylis*) have also been identified in sludge processes through microscopic observations (6). This previous study reported that the abundance of *Epistylis cf. rotans* positively correlated with ammonium removal efficiency. In addition, *Epistylis galea* is considered a consumer of CO_2_-assimilating microbes (*i.e.*, *Nitrosomonas* and *Nitrosococcus*) in activated sludge under ammonia-oxidizing conditions (26). Previous studies suggested that the abundance of *Epistylis* sp. weakly correlated with operation parameters such as effluent BOD, flow, DO, retained sludge concentration, SRT, sludge volume index (SVI), and Food/microorganisms (F/M) (18, 22).

We identified other shared OTUs in addition to the core and shared OTUs identified in the subclass Peritrichia (Table 3). Shared OTUs included close relatives to *Rhizidiomyces apophysatus* in the Stramenopiles, *Rhogostoma* sp. in the Rhizaria, *Lepadella rhombodies* in the Rotifera, and *Geotrichum fragrans* (in the phylum Ascomycota in Fungi), *Trichosporon cutaneum* (in the phylum Basidiomycota in Fungi), and the uncultured lineage LKM11 (in the phylum Cryptomycota in Fungi). Fungi have been overlooked by microscopic observations, and recent molecular surveys indicated that members of the Ascomycota, Basidiomycota, and Cryptomycota were the primary fungi in sewage treatment processes (9, 35). Members of the Ascomycota and Basidiomycota are capable of degrading cellulose, hemicellulose, and lignin (4, 24), and comprised 12 and 4%, respectively, of the total abundance of fungal clones in the libraries constructed in this study. These fungi may contribute to the degradation of cellulose, hemicellulose, and lignin in sewage because these materials comprise 2–8%, 9%, and 25%, respectively, of the total organic matter in sewage sludge in Japan (12, 14). In addition, *Trichosporon* sp. were shown to be involved in denitrification (13) and caused bulking at high DO levels (>2 mg L^−1^) (36).

Most of the fungal clones (78%) belonging to the LKM11 lineage in the phylum Cryptomycota and the OTUs belonging to the LKM11 lineage were shared in many of the libraries (Fig. 2, Table 3). This result is consistent with the study of Evans *et al.* (9), who investigated fungal diversity in activated sludge communities. The first representative sequence of the LKM11 lineage was retrieved from a freshwater engineered system and termed LKM11 by van Hannen *et al.* (34). Representative LKM11 sequences have been recovered from diverse environments, including soils, marine and freshwater sediments, freshwater planktonic samples, and oxygen-depleted environments. Organisms in the LKM11 lineage have been intensively investigated in terms of their phylogeny, ecology, and life cycles in freshwater environments (15, 17, 19). Although the functions of this uncultured lineage have not yet been defined, it is thought to be associated with the decomposition of detritus or phytoplanktonic organisms (microalgae and cyanobacteria) in oligotrophic and oligomesotrophic systems (19). Jones *et al.* (15) reported that some members of Cryptomycota in freshwater may be parasitic or saprotrophic. The functions of LKM11-lineage organisms in sewage treatment processes remain largely unknown; however, the recovery of diverse and numerous LKM11 sequences indicate that they most likely play an important role.

On the other hand, some eukaryotes that we expected to identify in the libraries were not found. Microscopic observations often identify amoebas in activated sludge (22, 37), but they were absent in our clone libraries. This bias was most likely caused by the methods we used for DNA extraction and/or PCR primers. In the present study, we employed a physical DNA extraction method (*i.e.*, bead-beating) to minimize the potential introduction of biases associated with enzymatic and chemical methods because eukaryotes have various cell structures. Regarding PCR primers, EukA and EukB, which are universal primers for *Eukarya*, were used in the present study because they can amplify nearly the full length of the 18S rRNA sequence. One of the reasons we selected the EukA-EukB primer set was to accumulate long and accurate 18S rRNA gene sequences because public databases only contain a very small number of sequences derived from eukaryotes in sewage treatment processes, which is important for future comparative eukaryotic community structural analysis using high-throughput sequencing technologies. The use of different primers may overcome this problem because Moreno *et al.* (26) detected a high fraction of Amoebozoa in the clone library of an activated sludge sample when using a different primer set.

In addition to the primer coverage issue, the presence of introns, which are rarely found in bacterial rRNA gene sequences, but are often present in eukaryotic sequences (2), is another concern when analyzing eukaryotes by molecular approaches. In this study, the length of several clones was high (*i.e.*, over 2,000 bp), and the results of BLAST searches indicated that these clones may not have been chimeric sequences. The length of the AOan_H_2012Dec_59 clone (accession number: AB901590) was 2,116 bases. The closest relative to this clone was *Tokophrya quadripartite* (AY102174), belonging to the Alveolata. It appears that the clone sequence has an insertion between bases 534 and 952 because it (except for the insertion region) shared 99% identity with that of *T. quadripartite*. A close relative of *T. quadripartite*, *T. lemnarum*, is known to contain an intron in the rRNA gene (11). An analysis of the transcripts, *i.e.*, rRNA, may be another option for exploring the molecular diversity of eukaryotes in environments.

## Conclusion

We herein sequenced a sufficient number of clones to explore the diversity of eukaryotes involved in sewage treatment processes. The results presented here showed that sewage treatment processes were characterized by a greater diversity of uncultured eukaryotes than was previously recognized. The dataset identified the core OTU in the subclass Peritrichia and several shared OTUs. The majority of the fungal sequences belonged to the LKM11 lineage in the phylum Cryptomycota; nevertheless, their ecology and roles in sewage treatment processes still remain unclear. The result that 60% of clones had <97% sequence identity to described eukaryotes indicates that deciphering the metabolic functions of these eukaryotes and estimating their contributions, especially to the degradation of cellulose and lignin, and nutrient removal, in sewage treatment are major directions for future research.

## Supplementary Information



## Figures and Tables

**Table 1:** Sewage treatment process operation conditions and performance characteristics

Process	Sample name	Flow rate (m^3^ d^−1^)	Temp. (°C)	DO (mg L^−1^)	Retained sludge		HRT (h)	SRT (d)	OLR (kgBOD m^3^ d^−1^)	BOD inf. (mg L^−1^)	BOD eff. (g-MLSS L^−1^)

					(g-MLVSS L^−1^)	(mg L^−1^)					
Activated sludge	AS_N_Sep	28,199	28.0	0.9	1.3	N.A.*	7.7	5.2	0.50	179	11
	AS_N_Dec	43,248	7.7	N.A.	1.0	0.8	8.7	7.0	0.69	161	14

	AS_S_Dec	106,247	16.2	1.2	1.8	1.4	5.6	5.8	0.36	170	5
	AS_K_Jan	65,100	19.0	0.8	2.0	1.7	5.5	6.5	0.61	218	5

Oxidation ditch	OD_Dec	2,187	17.5	N.A.	1.7	1.5	60	43	0.18	140	3

Two-step anoxic/oxic activated sludge	AO_an_Mar	15,866	13.3	N.A.	3.6	3.0	32	36	0.09	190	1
	AO_ox_Mar			0.01	3.8	3.2					

	AO_an_Dec	13,580	18.5	N.A.	2.9	2.5	38	36	0.08	205	1
	AO_ox_Dec			0.5	2.9	2.5					

* N.A.: Not available

**Table 2:** Eukaryotic community compositions and diversity indices based on 18S rRNA gene clone libraries

Process	Activated sludge				Oxidation ditch	Two-step anoxic/oxic activated sludge			

Sample name	AS_N_Sep	AS_N_Dec	AS_S_Dec	AS_K_Jan	OD_Dec	AO_an_Mar	AO_ox_Mar	AO_an_Dec	AO_ox_Dec
No. of analyzed clone	94	107	111	104	62	86	72	92	106
No. of OTU^a^	17	21	10	10	18	10	20	17	19
Coverage^b^	0.91	0.91	0.95	0.95	0.87	0.97	0.83	0.92	0.93
Evenness^b^	0.75	0.74	0.43	0.43	0.80	0.45	0.68	0.70	0.72
Chao1^b^	49	38	19	20	29	15	44	22	24

Fungi	**38.3**^c^	**48.6**		**10.6**	**21.0**	**10.5**	**16.7**	**70.7**	**71.7**
Euglenozoa	1.1		1.8	1.0	1.6	3.5	**13.9**	3.3	
Viridiplantae					3.2		1.4		
Alveolata	**12.8**	**28.0**	**96.4**	**11.5**	**41.9**	**81.4**	**56.9**	**19.6**	**25.5**
Stramenopiles	1.1	3.7	0.9		1.6			3.3	1.9
Rhizaria		5.6	0.9	**76.9**		1.2	4.2		
Amoebozoa							1.4		
Rotifera	1.1	**11.2**					1.4	2.2	0.9
Gastrotricha	6.4	0.9			**30.6**	3.5	4.2	1.1	
Nematoda	**39.4**	1.9							

a OTU was defined as ≥97% sequence identity as determined by mothur.

b Coverage index, Evenness, and the Chao1 nonparametric species richness estimator were calculated as described in Kubota *et al.* (16).

c Frequencies ≥10% are written in bold type.

**Table 3:** A list of eukaryotic OTUs shared among libraries and their fractions in each library. The number in each box represents the relative abundance of each OTU in each library

No.	AS_N_Sep	AS_N_Dec	AS_S_Dec	AS_K_Jan	OD_Dec	AO_an_Mar	AO_ox_Mar	AO_an_Dec	AO_ox_Dec	Kingdom/super phylum	phylum	Phylogenetic affiliation of close relatives	Accession No.	Seq. identiy (%)
	Relative abundance of each OTU (%)													
I		3			5	3	6		2	Fungi	Ascomycota	*Geotrichum fragrans*	AB000656	98–100
II	4			1	5				1	Fungi	Basidiomycota	*Trichosporon cutaneum*	AB001753	97–100
III	2	8						1	3	Fungi	—	uncultured (LKM11)	HM628670	97–99
IV	3	32		1		2	3			Fungi	—	uncultured (LKM11)	AY916571	99
V	10	3				3	1			Fungi	—	uncultured (LKM11)	AJ506016	98
VI	1	24		6	5	3	4	11	12	Alveolata	Ciliophora	*Epistylis chrysemydis*	AF335514	97–100
VII	10	1				77	49		5	Alveolata	Ciliophora	*Opercularia* sp.	AF401525	94
VIII	1	2		6	3					Alveolata	Ciliophora	uncultured (subclass Peritrichia)	JN020239	93–94
IX	1	1	1					3	2	Stramenopiles	—	*Rhizidiomyces apophysatus*	AF163295	97–98
X		5	1	77		1	4			Rhizaria	Cercozoa	*Rhogostoma* sp.	HQ121436	98–99
XI	1	11					1	2	1	Metazoa	Rotifera	*Lepadella rhomboides*	DQ297702	97–99
