# Supplementary material for: Molecular Diversity of Eukaryotes in Municipal Wastewater Treatment Processes as Revealed by 18S rRNA Gene Analysis
Source: Microbes Environ. 2014 Dec 10;29(4):401–7. doi: 10.1264/jsme2.ME14112 (PMC4262364; doi:10.1264/jsme2.ME14112)
Supplement: Supplementary file 1 [file 29_401_s1.pdf]

Supplementary Information

Molecular diversity of eukaryotes in municipal wastewater treatment processes as revealed by 18S rRNA gene analysis

Kengo Matsunaga, Kengo Kubota* and Hideki Harada
Department of Civil and Environmental Engineering, Tohoku University
6-6-06 Aza-Aoba, Aramaki, Aoba-ku, Sendai, Miyagi 980-8579 Japan

*Corresponding author:
Kengo Kubota,
Department of Civil and Environmental Engineering, Tohoku University,
6-6-06 Aoba, Aramaki, Aoba-ku, Sendai, Miyagi 980-8579 Japan
Tel & FAX: +81 (22) 795 5011
e-mail: kengok@epl1.civil.tohoku.ac.jp

Table S1 Water quality parameters of the samples used in this study.

Process	Sample name	pH (-)	TOC (mg/L)	CODcr (mg/L)	T-N (mg/L)	NH_4^+-N (mg/L)	NO_3^--N (mg/L)	NO_2^--N (mg/L)	T-P (mg/L)	PO_4^{3-}-P (mg/L)
Activated sludge	AS_N_Sep	6.8	145.0	36.5	34.4	11.6	11.8	0.4	4.6	4.0
	AS_N_Dec	6.8	18.3	17.3	8.4	7.6	0.8	0.1	0.1	0.1
	AS_S_Dec	7.2	23.2	23.1	17.9	17.4	<0.01	<0.01	3.0	0.4
	AS_K_Jan	7.3	33.4	38.6	30.7	30.4	<0.01	<0.01	14.4	11.0
Oxidation ditch	OD_Dec	6.9	16.7	34.7	2.7	2.2	0.3	0.0	1.1	0.8
Two-step anoxic/oxic activated sludge	AO_an_Mar	6.7	15.9	10.1	9.4	5.5	0.02	<0.01	3.6	0.2
	AO_ox_Mar	6.6	13.4	9.3	5.2	3.0	0.5	<0.01	1.0	0.1
	AO_an_Dec	6.9	18.1	19.3	8.9	8.0	0.02	<0.01	6.4	5.3
	AO_ox_Dec	6.6	17.4	17.3	5.0	3.7	1.1	0.0	1.9	1.6

* N.A : Not available

Table S2 List of operational taxonomic units (OTUs).

OUT No.	No. of clone	Kingdom/ Superphylum	phylum		acc. number of close relative	Distance matrix (%)	Accession No.
AS_N_Sep							
1	37	Metazoa	Nematoda	*Tobrilus gracilis*	AJ966506	99	AB901815
2	9	Alveolata	Ciliophora	subclass Peritrichia			AB901739
3	9	Fungi	Cryptomycota	LKM11			AB901723
4	9	Fungi	Cryptomycota	LKM11			AB901795
5	7	Fungi	-	-			AB901732
6	6	Metazoa	Gastrotricha	*Chaetonotus hystrix*	JQ798557	100	AB901816
7	4	Fungi	Basidiomycota	*Trichosporon laibachii*	AB001760	100	AB901769
8	3	Fungi	Cryptomycota	LKM11			AB901773
9	2	Fungi	Cryptomycota	LKM11			AB901763
10	1	Alveolata	Ciliophora	subclass Peritrichia			AB901786
11	1	Fungi	-	-			AB901791
12	1	Fungi	Cryptomycota	LKM15			AB901774
13	1	Alveolata	Ciliophora	subclass Peritrichia			AB901787
14	1	Alveolata	Ciliophora	class Phyllopharyngea			AB901784
15	1	Metazoa	Rotifera	*Lepadella rhomboides*	DQ297702	99	AB901809
16	1	Stramenopiles	-	*Rhizidiomyces apophysatus*	AF163295	97	AB901736
17	1	Euglenozoa	Euglenida	-			AB901779
AS_N_Dec							
1	34	Fungi	Cryptomycota	LKM11			AB902011
2	22	Alveolata	Ciliophora	*Epistylis chrysemydis*	AF335514	100	AB901979
3	11	Metazoa	Rotifera	*Lepadella rhomboides*	DQ297702	99	AB901965
4	9	Fungi	Cryptomycota	LKM11			AB901967
5	5	Rhizaria	Cercozoa	*Rhogostoma sp.*	HQ121436	99	AB901921
6	4	Alveolata	Ciliophora	subclass Peritrichia			AB902003
7	3	Fungi	Ascomycota	*Geotrichum fragrans*	AB000656	100	AB901996
8	3	Fungi	Cryptomycota	LKM11			AB901946
9	2	Alveolata	Ciliophora	subclass Peritrichia			AB902005
10	2	Stramenopiles	Oomycetes	order Peronosporales			AB902023
11	2	Metazoa	Nematoda	*Tobrilus gracilis*	AJ966506	99	AB901950
12	1	Rhizaria	Cercozoa	*Trinema enchelys*	AJ418792	99	AB901953
13	1	Stramenopiles	-	class Synurophyceae			AB901954
14	1	Fungi	Basidiomycota	*Itersonilia perplexans*	AB072228	100	AB902013
15	1	Alveolata	Ciliophora	subclass Peritrichia			AB902007
16	1	Fungi	Cryptomycota	LKM15			AB902006
17	1	Alveolata	Ciliophora	subclass Peritrichia			AB901933
18	1	Metazoa	Gastrotricha	*Chaetonotus hystrix*	JQ798557	100	AB902020
19	1	Metazoa	Rotifera	*Lecane elsa*	DQ297699	99	AB901980
20	1	Stramenopiles	-	*Rhizidiomyces apophysatus*	AF163295	97	AB901922
21	1	Fungi	Cryptomycota	LKM11			AB901956
AS_S_Dec							
1	75	Alveolata	Ciliophora	subclass Peritrichia			AB902108
2	26	Alveolata	Ciliophora	*Zoothamnium sp.*	DQ868356	97	AB902071
3	2	Alveolata	Ciliophora	*Tokophrya lemnarum*	AY332720	97	AB902135
4	2	Euglenozoa	Euglenida	-			AB902090
5	1	Alveolata	Ciliophora	subclass Peritrichia			AB902038
6	1	Stramenopiles	-	*Rhizidiomyces apophysatus*	AF163295	97	AB902100
7	1	Alveolata	Ciliophora	class Phyllopharyngea			AB902037
8	1	Alveolata	Ciliophora	subclass Peritrichia			AB902058

9	1	Alveolata	Ciliophora	subclass Peritrichia			AB902048
10	1	Rhizaria	Cercozoa	*Rhogostoma sp.*	HQ121436	98	AB902057
AS_K_Jan							
1	80	Rhizaria	Cercozoa	*Rhogostoma sp.*	HQ121436	98	AB901847
2	6	Alveolata	Ciliophora	subclass Peritrichia			AB901917
3	6	Alveolata	Ciliophora	subclass Peritrichia			AB901881
4	4	Fungi	Cryptomycota	LKM11			AB901898
5	3	Fungi	Cryptomycota	LKM11			AB901859
6	1	Fungi	Basidiomycota	*Trichosporon cutaneum*	AB001753	100	AB901822
7	1	Fungi	Cryptomycota	LKM11			AB901883
8	1	Fungi	Cryptomycota	LKM11			AB901858
9	1	Fungi	Ascomycota	*Galactomyces geotrichum*	GQ458033	100	AB901846
10	1	Euglenozoa	Euglenida	-			AB901832
OD_Dec							
1	19	Metazoa	Gastrotricha	*Chaetonotus daphnes*	JQ798549	100	AB902310
2	13	Alveolata	Ciliophora	class Phyllopharyngea			AB902305
3	4	Alveolata	Ciliophora	class Phyllopharyngea			AB902319
4	3	Fungi	Ascomycota	*Candida sp.*	AY520174	98	AB902335
5	3	Fungi	Basidiomycota	*Trichosporon cutaneum*	AB001753	100	AB902356
6	3	Alveolata	Ciliophora	*Epistylis chrysemydis*	AF335514	97	AB902312
7	3	Fungi	Ascomycota	*Geotrichum klebahnii*	AB000641	100	AB902349
8	2	Alveolata	Ciliophora	class Phyllopharyngea			AB902341
9	2	Alveolata	Ciliophora	subclass Peritrichia			AB902358
10	2	Viridiplantae	Chlorophyta	-			AB902329
11	1	Fungi	Ascomycota	class Saccharomycetes			AB902354
12	1	Euglenozoa	Euglenida	-			AB902317
13	1	Fungi	Ascomycota	class Laboulbeniomycetes			AB902342
14	1	Alveolata	Ciliophora	class Plagiopylea			AB902303
15	1	Stramenopiles	Oomycetes	*Lagenidium giganteum*	M54939.1	98	AB902347
16	1	Fungi	Ascomycota	*Dipodascus capitatus*	AB000650	100	AB902344
17	1	Fungi	Ascomycota	class Saccharomycetes			AB902298
18	1	Alveolata	Ciliophora	class Phyllopharyngea			AB902321
AO_an_Mar							
1	66	Alveolata	Ciliophora	subclass Peritrichia			AB902282
2	3	Alveolata	Ciliophora	subclass Peritrichia			AB902248
3	3	Fungi	Ascomycota	*Geotrichum fragrans*	AB000656	100	AB902274
4	3	Metazoa	Gastrotricha	*Polymerurus nodicaudus*	JN185490	97	AB902226
5	3	Euglenozoa	Euglenida	-			AB902245
6	3	Fungi	Cryptomycota	LKM11			AB902291
7	2	Fungi	Cryptomycota	LKM11			AB902294
8	1	Rhizaria	Cercozoa	*Rhogostoma sp.*	HQ121436	99	AB902257
9	1	Fungi	Ascomycota	-			AB902263
10	1	Alveolata	Ciliophora	class Phyllopharyngea			AB902221
AO_ox_Mar							
1	35	Alveolata	Ciliophora	subclass Peritrichia			AB902186
2	9	Euglenozoa	Euglenida	-			AB902203
3	4	Fungi	Ascomycota	*Geotrichum fragrans*	AB000656	99	AB902177
4	3	Rhizaria	Cercozoa	*Rhogostoma sp.*	HQ121436	99	AB902189
5	3	Alveolata	Ciliophora	*Epistylis chrysemydis*	AF335514	98	AB902204
6	2	Alveolata	Ciliophora	*Tokophrya quadripartita*	AY102174	97	AB902200
7	2	Fungi	Cryptomycota	LKM11			AB902187
8	2	Metazoa	Gastrotricha	*Polymerurus nodicaudus*	JN185490	97	AB902151

9	1	Fungi	Cryptomycota	LKM11			AB902172
10	1	Fungi	Cryptomycota	LKM11			AB902148
11	1	Fungi	Ascomycota	*Galactomyces geotrichum*	X69842	99	AB902190
12	1	Amoebozoa	Flabellinea	-			AB902166
13	1	Alveolata	-	-			AB902201
14	1	Euglenozoa	Euglenida	-			AB902142
15	1	Metazoa	Gastrotricha	*Lepidodermella squamata*	U29198	99	AB902207
16	1	Metazoa	Rotifera	*Lecane elsa*	DQ297699	99	AB902195
17	1	Fungi	Cryptomycota	LKM11			AB902208
18	1	Fungi	Ascomycota	*Galactomyces geotrichum*	JQ698930	99	AB902196
19	1	Fungi	Ascomycota	*Candida sake*	AB013529	100	AB902141
20	1	Viridiplantae	Chlorophyta	-			AB902205
AO_an_Dec							
1	31	Fungi	Cryptomycota	LKM11			AB901565
2	27	Fungi	Cryptomycota	LKM11			AB901556
3	10	Alveolata	Ciliophora	*Epistylis chrysemydis*	AF335514	97	AB901564
4	4	Alveolata	Ciliophora	class Phyllopharyngea			AB901590
5	3	Stramenopiles	-	*Rhizidiomyces apophysatus*	AF163295	98	AB901553
6	2	Alveolata	Ciliophora	-			AB901611
7	2	Fungi	Ascomycota	-			AB901576
8	2	Metazoa	Rotifera	*Lecane leontina*	DQ297700	99	AB901579
9	2	Euglenozoa	Euglenida	-			AB901604
10	2	Alveolata	Ciliophora	subclass Peritrichia			AB901562
11	1	Fungi	Ascomycota	-			AB901547
12	1	Fungi	Cryptomycota	LKM11			AB901574
13	1	Fungi	Cryptomycota	LKM15			AB901587
14	1	Metazoa	Gastrotricha	*Polymerurus nodicaudus*	JN185490	99	AB901583
15	1	Fungi	Ascomycota	class Saccharomycetes			AB901531
16	1	Fungi	Ascomycota	class Laboulbeniomycetes			AB901584
17	1	Euglenozoa	Euglenida	-			AB901616
AO_ox_Dec							
1	37	Fungi	Cryptomycota	LKM11			AB901667
2	25	Fungi	Cryptomycota	LKM11			AB901672
3	11	Alveolata	Ciliophora	subclass Peritrichia			AB901721
4	5	Alveolata	Ciliophora	*Tokophrya quadripartita*	AY102174	97	AB901715
5	5	Alveolata	Ciliophora	subclass Peritrichia			AB901706
6	3	Fungi	Cryptomycota	LKM15			AB901699
7	3	Fungi	Cryptomycota	LKM11			AB901634
8	2	Alveolata	Ciliophora	subclass Peritrichia			AB901620
9	2	Fungi	Ascomycota	*Geotrichum fragrans*	AB000656	100	AB901716
10	2	Fungi	Chytridiomycota	-			AB901696
11	2	Stramenopiles	-	*Rhizidiomyces apophysatus*	AF163295	97	AB901664
12	2	Alveolata	Ciliophora	subclass Peritrichia			AB901670
13	1	Metazoa	Rotifera	*Lecane elsa*	DQ297699	99	AB901717
14	1	Alveolata	-	-			AB901649
15	1	Fungi	Basidiomycota	*Trichosporon cutaneum*	AB001753	100	AB901638
16	1	Fungi	Cryptomycota	LKM11			AB901682
17	1	Fungi	Ascomycota	-			AB901684
18	1	Fungi	Ascomycota	-			AB901641
19	1	Alveolata	Ciliophora	subclass Peritrichia			AB901648

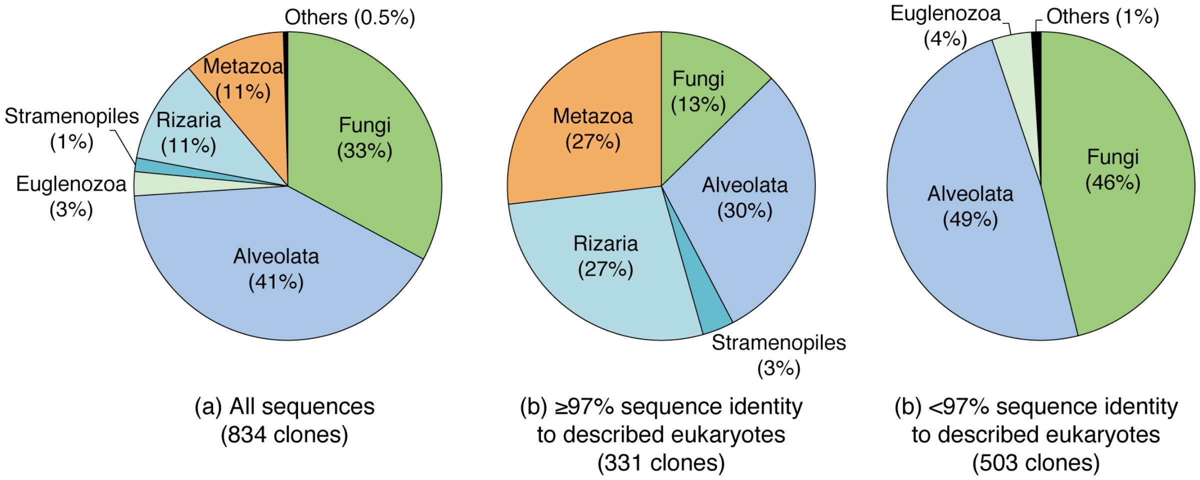

Fig. S1. Kingdom/superphylum-level eukaryotic community composition of 834 clones (a). Eukaryotic compositions based on clones with ≥97% (b) or <97% (c) sequence identity to described eukaryotes.

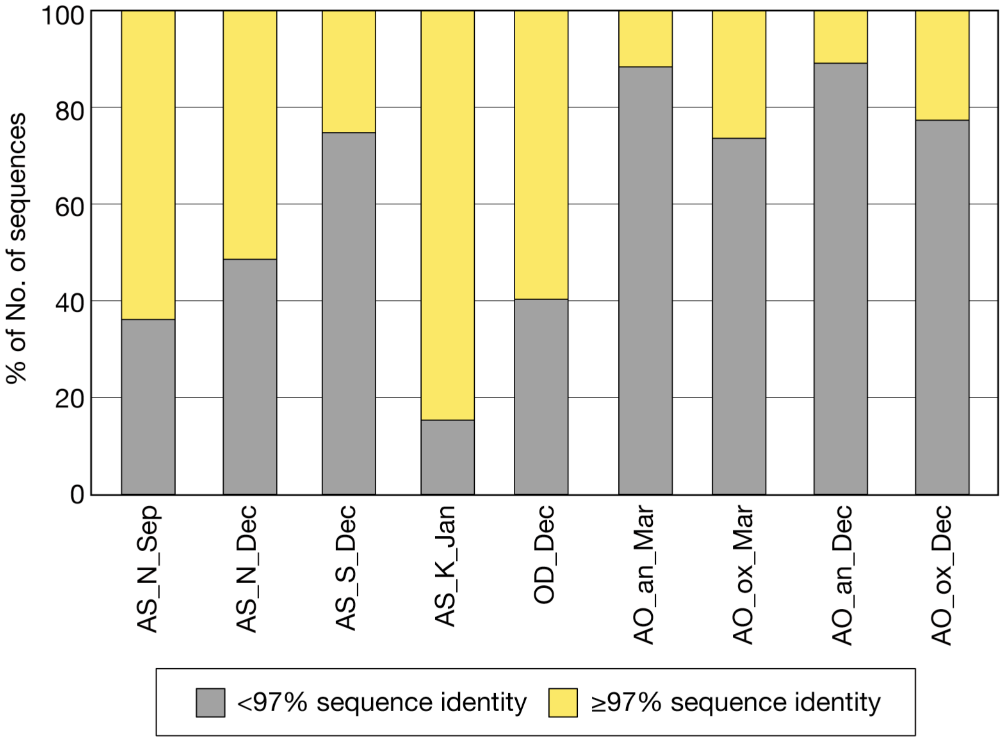

Fig. S2. Fraction of clones with <97% and ≥97% sequence identities to described eukaryotes in each clone library.

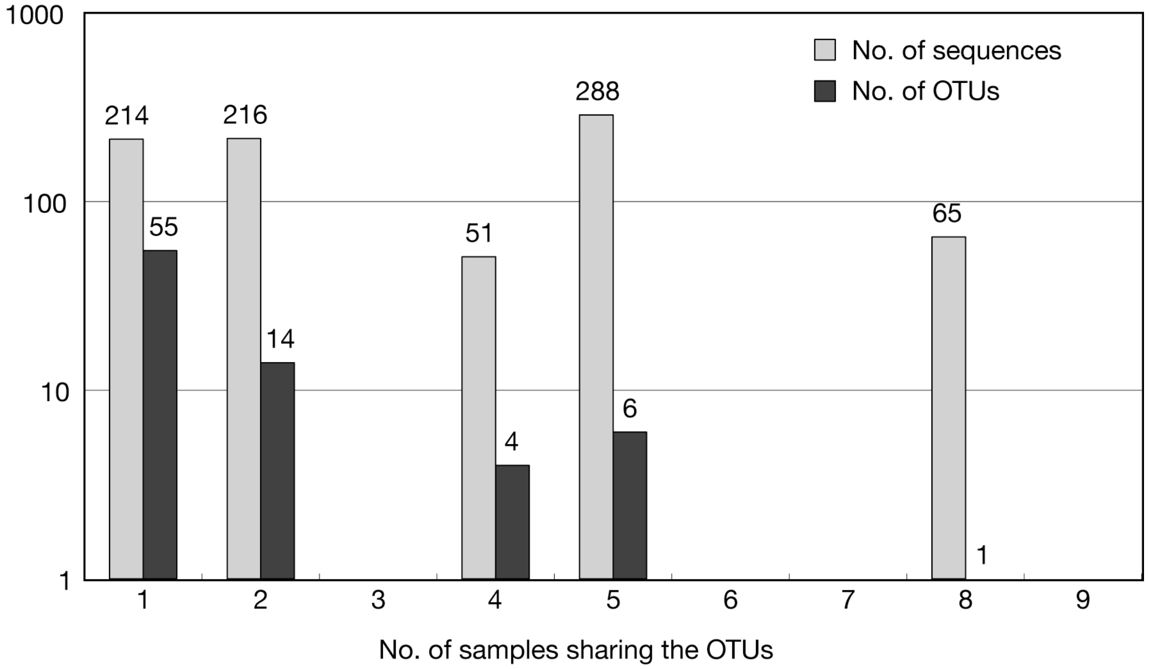

Fig. S3. Distribution of shared operational taxonomic units (OTUs) according to the number of samples sharing the OTU.
